# Clinical activity and safety of sintilimab, bevacizumab, and TMZ in patients with recurrent glioblastoma

**DOI:** 10.1186/s12885-024-11848-z

**Published:** 2024-01-25

**Authors:** Yinghao Lu, Limin Liao, Kunpeng Du, Jianhua Mo, Xia Zou, Junxian Liang, Jiahui Chen, Wenwen Tang, Liwei Su, Jieping Wu, Junde Zhang, Yujing Tan

**Affiliations:** 1 Department of Radiation Oncology, Zhujiang Hospital, Southern Medical University, No 253, Gongye Road, Guangzhou, 510280 China; 2 Department of Image, Zhujiang Hospital, Southern Medical University, Guangzhou, 510280 China; 3 Department of Surgery, Zhujiang Hospital, Southern Medical University, Guangzhou, 510280 China

**Keywords:** Recurrent glioblastoma, Immunotherapy, Sintilimab, Treatment outcomes

## Abstract

**Purpose:**

There are limited and no standard therapies for recurrent glioblastoma. We herein report the antitumour activity and safety of sintilimab, bevacizumab and temozolomide (TMZ) in recurrent glioblastoma.

**Methods:**

We retrospectively analysed eight patients with recurrent glioblastoma treated with sintilimab (200 mg) every three weeks + bevacizumab (10 mg/kg) every three weeks + TMZ (200 mg/m²orally) (5 days orally every 28 days for a total of four weeks). The primary objective was investigator-assessed median progression-free survival(mPFS). Secondary objectives were to assess the 6-month PFS, objective response rate (ORR) and duration of response (DOR) accroding to RANO criteria.

**Results:**

The mPFS time for 8 patients was 3.340 months (95% CI: 2.217–4.463), The longest PFS was close to 9 months. Five patients were assessed to have achieved partial response (PR), with an overall remission rate of 62.5%, Four patients experienced a change in tumour volume at the best response time of greater than 60% shrinkage from baseline, and one patient remained progression free upon review, with a DOR of more than 6.57 months. The 6-month PFS was 25% (95% CI: 5.0–55.0%). Three patients had a treatment-related adverse events, though no grade 4 or 5 adverse events occurred.

**Conclusion:**

In this small retrospective study, the combination regimen of sintilimab, bevacizumab and TMZ showed promising antitumour activity in treatment of recurrent glioblastoma, with a good objective remission rate.

## Introduction

Glioblastoma is the most common malignant primary brain tumour in adults, and with aggressive treatment, surgery, radiotherapy and TMZ, the median overall survival is approximately 14.6 months [1]; the median overall survival is estimated to be 24–44 weeks [2, 3]. At present, there is no single standard treatment plan for recurrent high-grade glioma. Previous studies have indicated that the growth and recurrence of high-grade glioma are closely related to tumour angiogenesis. As a humanized monoclonal antibody against VEGF, bevacizumab has been approved for use in treating recurrent high-grade gliomas in China and abroad. This approval is based on data from an early phase II in which improvement in PFS was observed but no improvement in OS [4, 5]. Although single agent bevacizumab is approved for recurrent GBM, the combination of antiangiogenic therapies with cytotoxic chemotherapy agents remains a commonly used strategy for many cancers, including colon cancer. including but not limited to TMZ and irinotecan, have not significantly improved on the prior benchmark of 42.6% for PFS6, with PFS6 for these studies ranging from 6.7 to 50.3% [5–11]. Hence, there is a need to expand the understanding of the role of BEV in recurrent GBM and to investigate other new BEV regimens.

Immune checkpoint inhibitors are BEV partners compound worth considering, and while single-agent immune checkpoint inhibitors have not yet yielded promising results in recurrent GBM, preoperative use of nabulizumab combined with continuation of therapy postoperatively provided long-term survival benefits for two patients with newly diagnosed neuroblastoma, who survived 33 and 28 months postoperatively [12]. Sintilimab, a domestically produced human immunoglobulin G4 (IgG4) monoclonal antibody (HuMAb), binds to programmed cell death receptor 1 (PD-1) expressed on T cells, blocking its interaction with PD-L1. This interruption inhibits the PD-1 pathway-mediated immune-suppressive response, including anti-tumor immune reactions. Sintilimab received expanded medical insurance indications after adapting to relapsed or refractory classical Hodgkin lymphoma, gaining approval for first-line treatment in non-squamous, squamous non-small cell lung cancer, and unresectable or metastatic hepatocellular carcinoma. This expansion underscores the safety and effectiveness of sintilimab. Compared with similar drugs, sintilimab, has high affinity and durable stability, high saturated receptor occupancy, and strong stimulation of T-cell activation compared to similar drugs, which significantly prolonging patient survival in advanced squamous lung, oesophageal, and hepatocellular carcinomas, among others [13–15]. Although sintilimab currently lacks indications in gliomas and lacks clinical research data, given the non-beneficial outcomes of nivolumab and camrelizumab in glioblastoma studies, we choose to explore the investigational use of sintilimab based on these four major advantages. In addition, the combination of antiangiogenic drugs and immunosuppressive agents has been shown to alter the bidirectional regulation of angiogenic factors and suppress immune cells. TMZ has been shown to cause a decrease in lymphocytes and an increase in the proportion of Tregs, and potentially enhance dendritic cell function. Considering the synergistic effects of immunosuppressive agents with chemical and antiangiogenic drugs, we retrospectively analysed the efficacy and safety of the three-agent combination of TMZ, bevacizumab, and sintilimab for treatment of recurrent glioma.

## Materials and methods

### Study design and patients

We retrospectively analysed 8 patients with first or second recurrence of glioblastoma confirmed by resurgery or imaging between 2022-01-20 and 2023-04-13 who were treated orally according to the following regimen: sintilimab 200 mg every three weeks + bevacizumab 10 mg/kg every three weeks + TMZ 200 mg/m² orally for 5 days every 28 days for four weeks, until the patient experienced tumour progression, worsening clinical symptoms or unacceptable toxicity. The primary objective was investigator-assessed median progression-free survival (mPFS), namely, time from baseline to the first recorded progressive disease (PD) or early death from any cause, whichever occurred first. The secondary objective was to assess the 6-month PFS, ORR and DOR(the time from when subjects first achieved a partial response to the time of first disease progression or death from any cause ) per RANO criteria. mPFS was analysed by Kaplan-Meier analysis and is reported with 2-sided 95% CIs. Diverse reactions occurring during treatment were analysed based on reported TRAEs, which were graded according to CTCAE v5.0.

### Radiographic evaluation

Patients underwent baseline MRI within 2 weeks prior to starting treatment, and cranial MRI was reviewed every two or three dosing cycles. The patients were assessed for PD, SD or PR according to the Response Assessment in RANO criteria of imaging changes: PR considered a reduction of more than 50% in the sum of the products of the two vertical diameters of the tumour under the largest cross-section compared to baseline; PD an increase of more than 25% in the sum of the products of the two vertical diameters of the tumour under the largest cross-section; and SD between PD and PR.

### Immunohistochemistry (IHC)

Paraffin-embedded tissue specimens were cut into 4 μm-thick sections (purchased from the Department of Pathology, Zhujiang Hospital, Southern Medical University). The sections were incubated at 68 °C for 1.5 h, dewaxed in xylene and rehydrated in an alcohol gradient. Antigen retrieval was performed using sodium citrate buffer (Solebo, China) at 95–100 °C for 8 min, after which the sections were cooled to room temperature. The sections were then washed three times with PBS for 5 min each, after which endogenous peroxidase activity was blocked with 3% hydrogen peroxide (Beyotime, China) for 15 min. Next, nonspecific binding sites were blocked with 5% goat serum (Boster, China) at room temperature for 30 min. Subsequently, the sections were incubated overnight at 4 °C with a specific primary antibody against PD-L1 (1:200; HUABIO, China). On the following day, the sections were washed three times with PBS for 5 min each, followed by a 30-minute incubation at room temperature with secondary antibodies. HRP activity was detected using 3,3’-diaminobenzidine (DAB) (Dako, Denmark), and cell nuclei were stained with haematoxylin. Finally, the sections were dehydrated in a gradient of an alcohol gradient and cleared in xylene before being mounted with neutral resin. Image acquisition was performed using a Leica DM2500 microscope.

### IHC evaluation

PD-L1 expression after staining of slides was observed via microscopy. A percentage of tumour cells with cytoplasmic or cell membrane staining was considered positive for PD-L1 expression. Two pathologists separately examined PD-L1 expression, with more than 90% concordance.

## Results

### Patients

We included eight patients with glioblastoma recurrence from 2022-01-20 to 2023-04-13, two (25%) of whom had oligodendroglioma secondary to glioblastoma. Four (50%) patients were female, with a median age at recurrence of 57.5 years (range from 43 to 74). Five patients were treated after recurrence (including recurrence after diagnosis of glioblastoma): one underwent surgery, two were treated with bevacizumab (5–15 mg/kg every 2–3 weeks) monotherapy, and two participated in a clinical trial (with the GNC-039 tetraspecific antibody). At baseline, 2 patients were receiving steroids for symptoms associated with cerebral oedema. Archived tumour specimens were obtained for all 7 patients, and one patient could not be accessed without surgical intervention after diagnosis of secondary glioblastoma. Patient data regarding the status of the tumour promoter O6-methylguanine-DNA methyltransferase (MGMT) were collected, and one of the seven patients was found to have an MGMT-methylated tumour. Immunohistochemistry at recurrence of glioblastoma diagnosis in a patient with progression to oligodendroglioma suggested a shift from mutant IDH to wild-type IDH. Thus, except for one patient whose oligodendroglioma progressed to glioblastoma who did not undergo surgical resection or pathological testing at the time of recurrence, all seven patients had wild-type IDH. The patient baseline demographic data are listed in Table 1.

**Table 1:** Patient characteristics

Patient charactiristics(*n* = 8)	NO. (%)	All patient (%)
**PRS.type**		
Primary	6(75%)	8(100%)
secondary	2(25%)	8(100%)
**History**		
GBM	8(100%)	8(100%)
**Grade**		
WHO IV	8(100%)	8(100%)
**Gender**		
male	4(50%)	8(100%)
Female	4(50%)	8(100%)
**Age**		
40–50	2(25%)	8(100%)
50–60	3(37.5%)	8(100%)
≥ 60	3(37.5%)	8(100%)
**PD-L1**		
<1%	7(87.5%)	8(100%)
≥ 1%	1(12.5%)	8(100%)
**Ki−67**		
0–20	2(28.6%)	7(87.5%)
≥ 20	5(71.4%)	7(87.5%)
**P53**		
wildtype	4(57.1%)	7(87.5%)
Mutant	3(42.9%)	7(87.5%)
**IDH−1**		
wildtype	7(100%)	7(87.5%)
Mutant	0	7(87.5%)
**MGMTp.methylation.status**		
Methylated	1(14.3%)	7(87.5%)
Unmethylated	6(85.7%)	7(87.5%)
**1p.19q.codeletion.status**		
Non-codel	2(100%)	2(25%)
codel	0	2(25%)
**No. of recurrences**		
1	3(37.5%)	8(100%)
2	5(62.5%)	8(100%)
**prior therapy**		
Surgery	1(20%)	5(62.5%)
Chemotherapy	4(80%)	5(62.5%)
Radiation	0	4(50%)
**Corticosteroid use at baseline**		
Yes	2(25%)	8(100%)
No	6(75%)	8(100%)
**Treatment-related AEs**		
all grade	3(37.5%)	8(100%)
Grade 3 treatment-related AEs	1(33.3%)	8(100%)
Grade 1–2 treatment-related AEs	3(37.5%)	8(100%)

Abbreviation: *AEs *Adverse events

**Table 2:** Patient characteristics and outcome

No.	Gender	History	PRS-type	Prior therapy	Dose of bevacizumab	Dose of TMZ	Dose of Sintilimab	Using period	Best response	PFS(months)	DOR(months)
1	M	GBM	Primary	Surgery	200(3) 500(2)	160	200	5	PR	3.29	1.84
2	M	GBM	secondary	NA	500	240	200	4	PR	2.70	1.27
3	M	GBM	Primary	Bevacizumab	500	300	200	8	PR	6.10	5.16
4	F	GBM	Primary	Bevacizumab	600	300	200	4	SD	4.10	
5	M	GBM	Primary	GNC−039	500	300	200	7	SD	5.10	
6	F	GBM	Primary	NA	500	260	200	10	PR	8.52+	6.57+
7	F	GBM	Primary	GNC−039	500	240	200	5	PR	3.34	1.58
8	F	GBM	secondary	NA	600	240	200	3	PD	1.45	

*Abbreviations*: *TMZ *Temozolomide, *PR *Partial response, *SD *Stable disease, *PD *Progressive disease, *mPFS *Median progression-free survival, *ORR *Overall response rate, *DOR *Duration of Response

No.1 patient was on 200 mg bevacizumab for the first three cycles, and due to worsening clinical symptoms, the dose was increased to 500 mg for the last two cycles

**Fig. 1:**
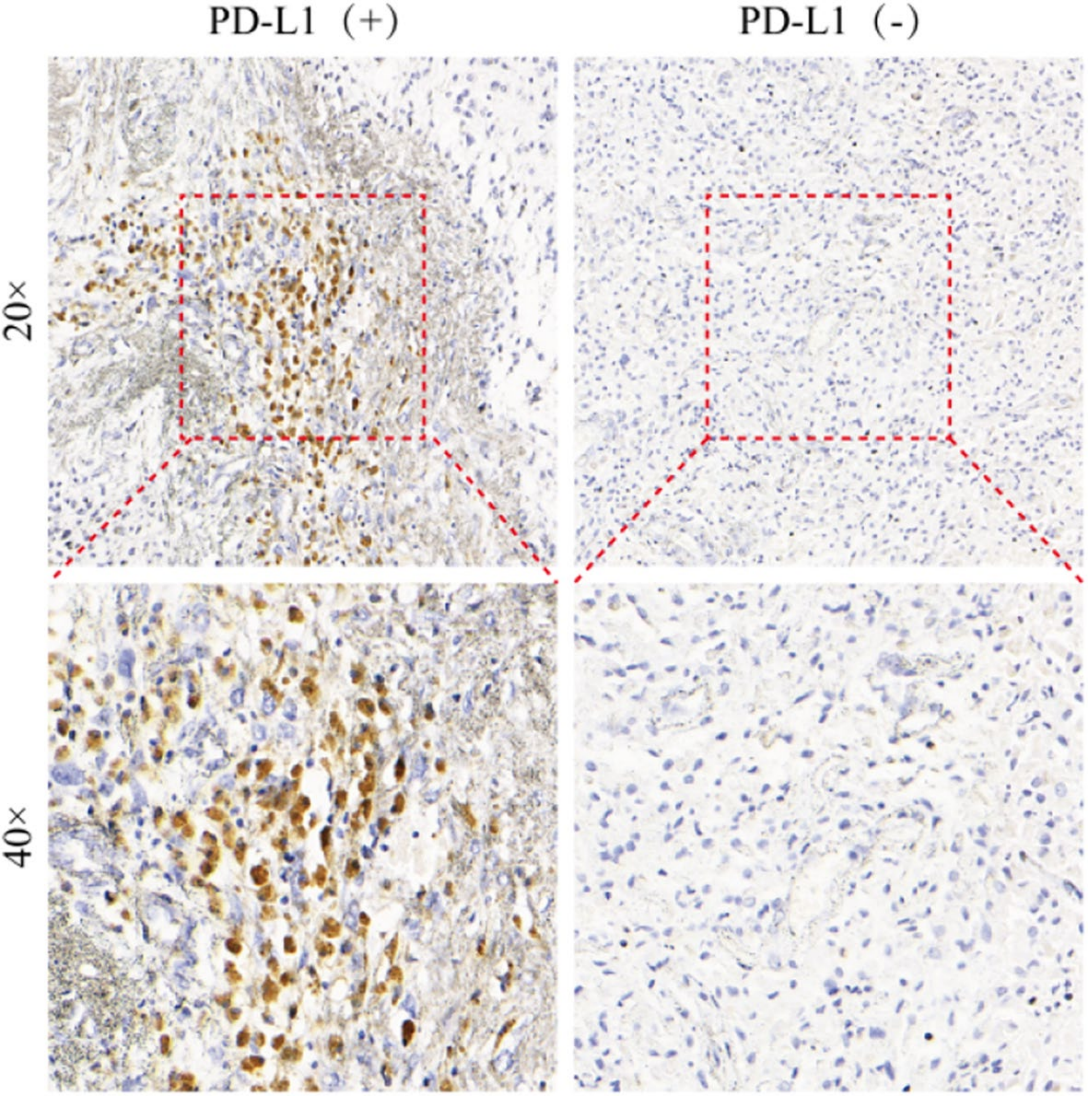
Expression of PD-L1 after staining of slides by microscopy, comparison of PD-L1-positive and PD-L1-negative tissue specimens

To determine whether PD-L1 expression has any impact on prognosis, we performed immunohistochemical staining on the 6 patients whose postoperative pathology did not involve testing for PD-L1 (paraffin specimens were unavailable for one of these patients). Immunohistochemical results (integrating data from 2 patients who had undergone PD-L1 testing) revealed that 5 patients were negative for PD-L1 expression and that 1 patient had more than 1% PD-L1-stained cells (Fig. 1).

### Efficacy

The investigator-assessed ORR according to RANO criteria was 62.5%, with 5 of the 8 patients achieving PR. One patient’s insufficient dosage of bevacizumab during the first three cycles of treatment did not affect that assessment of efficacy, as PR occurred in the second cycle of treatment. All five patients with PR had a DOR of more than one month, two patients had a DOR of more than five months, and one patient still had not experienced progression on review, with a DOR of more than 6.57 months (Table 2).

**Fig. 2:**
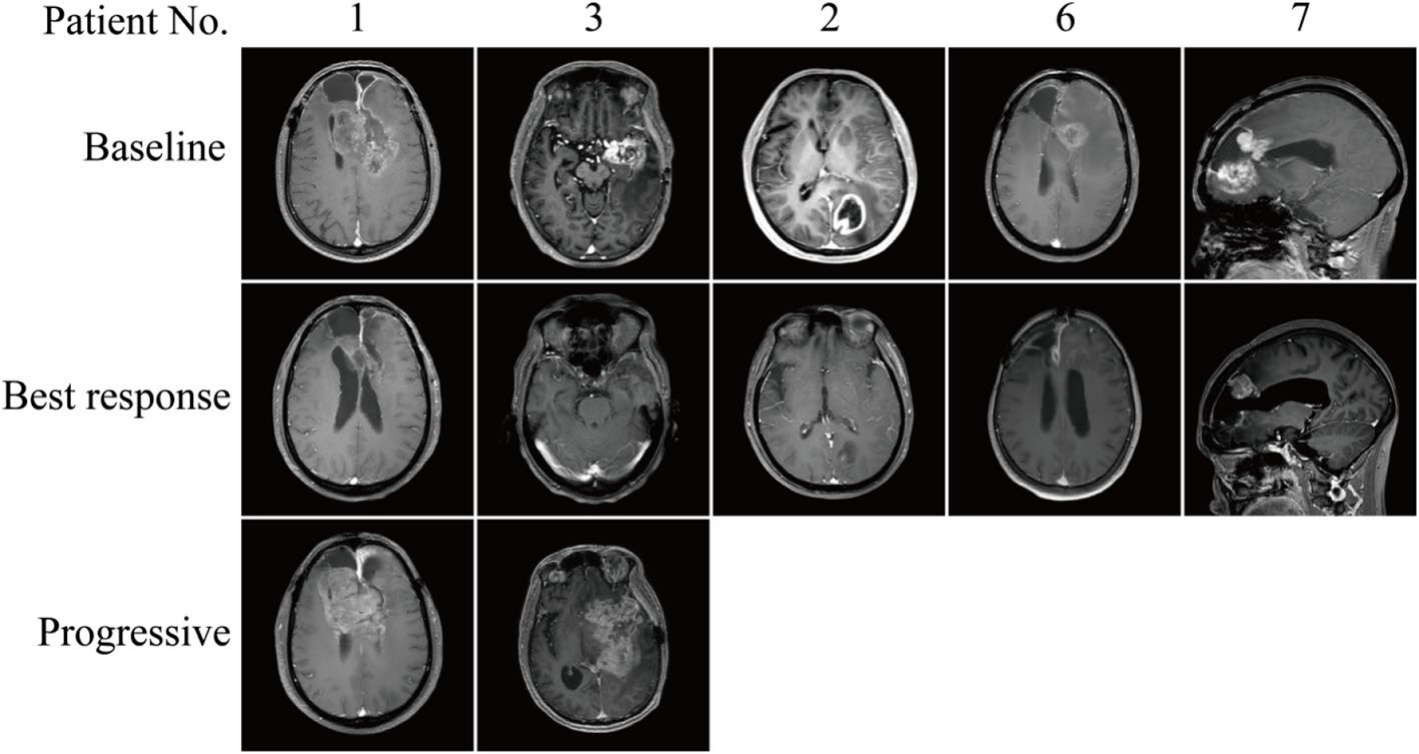
MR images(all T1-weighted images)at baseline, at the time of optimal efficacy and at the time of progression in five patients who achieved partial remission (MR at the time of relapse was not recorded in three of these patients due to loss to follow-up)

**Fig. 3:**
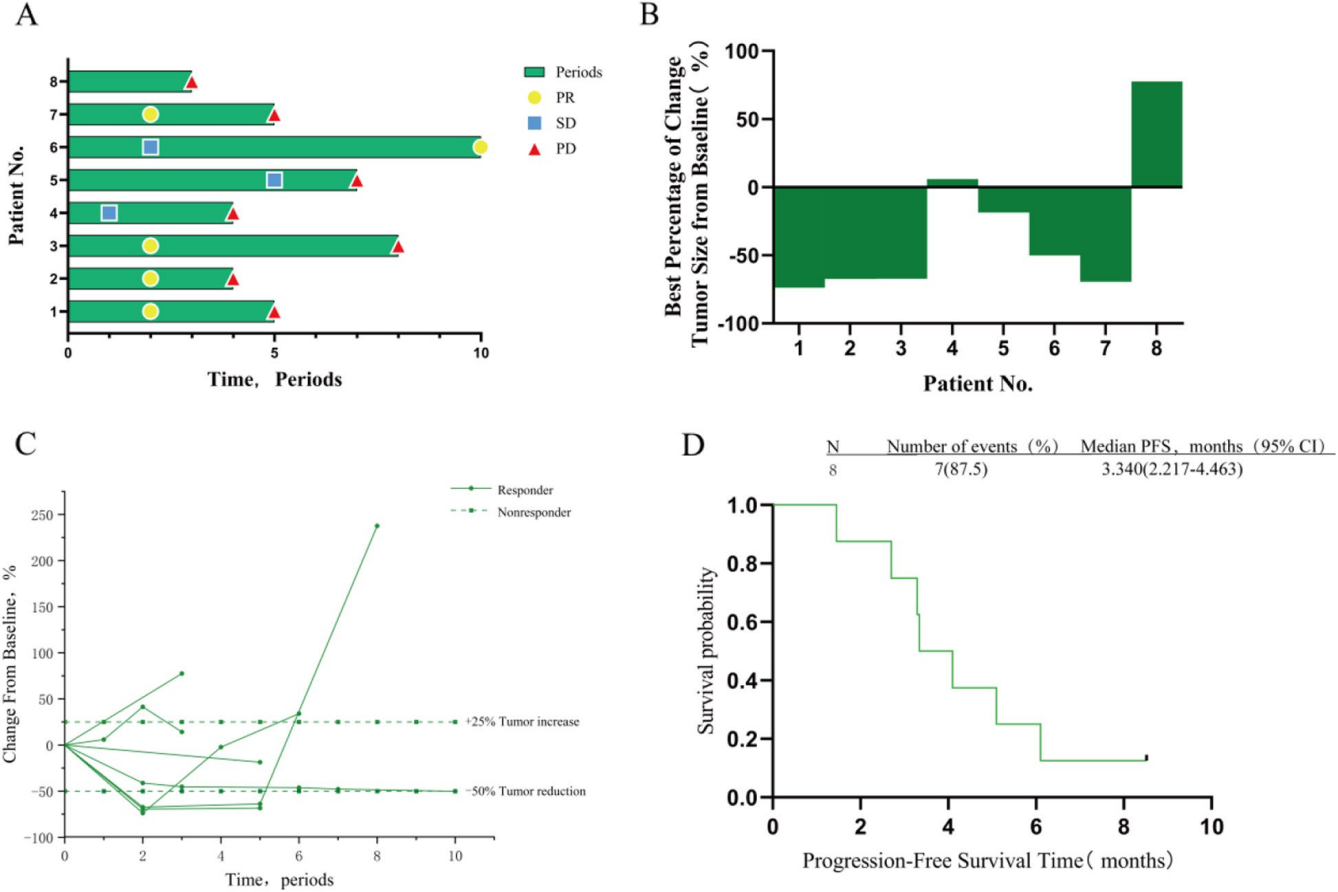
Treatment exposure and responses are illustrated in individual patients based on RANO criteria. Only patients who had ≥1 evaluable postbaseline tumor assessment are included (*n* = 8). **A** Treatment and response duration, including confirmed and unconfirmed responses, are illustrated. **B **Tumor volume compared to baseline when patients achieve best efficacy during treatment. **C** Longitudinal percentage change from baseline in tumor size are illustrated. **D **Survival estimates are illustrated in patients who received ≥1 dose of Sintilimab, Bevacizumab, and Temozolomide including progression-free survival (PFS) based on RANO criteria

Patient efficacy was assessed using RANO criteria, and five of the eight patients achieved PR according to efficacy assessment (Fig. 2), with four patients found on review after two cycles of the drug (Fig. 3A). The change in tumour volume at the time of best response was greater than 60% of baseline in all four patients, with the patient with the best efficacy showing 73.8% tumour regression (Fig. 3B). One patient did not return for review due to the epidemic, and only the MR based on his five-cycle review on the drug was obtained; tumour shrinkage rate was 18.6% from baseline, which was considered to indicate SD. The tumour in one patient initially increased in size, subsequently decreased, but consistently remained larger than the baseline, indicating a gradual progression. Given that over two years have passed since the patient underwent radiotherapy at the initial diagnosis, and considering the absence of signs of inflammation, edema, or transient blood-brain barrier permeability causing localized enhancement on magnetic resonance imaging, and with the patient having received bevacizumab and temozolomide in previous treatments, following multidisciplinary consultation, we do not interpret the tumour enlargement in this patient post-combination therapy as indicative of pseudoprogression. Instead, we consider it as a true progression of the tumour, possibly due to the delayed effectiveness of the three-drug combination therapy. The other patient’s tumour reached PD after 2 cycles of the drug and was considered nonresponsive to the triple-drug combination treatment (Fig. 3C). Because the patient’s time to tumour progression was less than 2 months and the tumour load had increased by more than 50% compared to baseline, it is reasonable to evaluate if hyperprogression occurred in this patient. Another patient showed 41.2% tumour regression from baseline at the time of review after 2 cycles of the drug, the tumour volume continued to decrease afterwards, and the tumour efficacy reached PR by the time of review after 10 cycles of the drug. No correlation between baseline corticosteroid use and poor prognosis was observed. The median PFS for the eight patients was 3.340 months (95% CI: 2.217–4.463). Three of the patients had PFS of more than 5 months (Fig. 3D). The longest PFS was close to 9 months. The 6-month PFS was 25% (95% CI: 5.0–55.0%)

Three (37.5%) of the patients developed treatment-related AEs, all grade 1 TRAEs (leukopenia); one patient had grade 3 TRAEs (elevated alanine aminotransferase (ALT)), which was treated with hepatoprotective medications and returned to normal values; and one patient had asymptomatic elevation of TSH. None of these patients discontinue triple-drug combination therapy due to treatment-related AEs (Table 1).

## Discussion

Glioblastoma has historically been considered a difficult disease to treat, and treatment options for recurrent glioma are limited. Our innovative three-agent combination antitumour regimen of TMZ + bevacizumab + sintilimab was retrospectively analysed in eight patients with recurrent glioblastoma, with a median PFS of 3.340 months (95% CI: 2.217–4.463). The preliminary median PFS data were consistent with those of other treatments in recurrent glioma patients, with an ORR of 62.5%. This figure rarely exceeded 50% in previous clinical studies of combination therapy applied to recurrent glioma, and more promisingly, the longest DOR for 5 patients with PR was close to 9 months. One patient still had not experienced progression at the time of review. Since the seven patients with stable or partially remitted disease were treated concurrently with the three-drug combination, it is difficult to attribute stable disease control to use of one drug alone.

After several years of research, PD-1 monoclonal antibody blockers have shown promising results in patients with advanced cancer [16]. However, there is limited success in treating glioma with single-agent immunosuppressive therapy. A prospective study of recurrent glioma patients treated with atezolizumab monotherapy enrolled 16 patients with an ORR of 6% and mPFS of 1.2 months (range 0.7–10.7 months) [17]. Another phase study of pembrolizumab for recurrent glioma enrolled 26 PD-L1-positive patients with mPFS of 2.8 months and ORR of 8%. It was not possible to determine the relationship between PD-L1 expression and immunosuppressant efficacy in this study because all the enrolled patients were PD-L1 positive [18]. Possible reasons for the failure of anti-PD-1 immunosuppressant monotherapy include radiotherapy-induced lymphopaenia and the unresponsiveness of effector T cells to tumour-specific antigens in the TME. In addition, Wherry et al. examined the phenotype of tumour-infiltrating lymphocytes (TILs) in glioma specimens and found that they were enriched in CD95, D-1, PD-L1, CTLA-4, LAG3, and TIM-3, which clearly suggests immune depletion of T cells [19]. There is evidence that GBM patients with pretreatment immunoreactivity at the tumour site may have a favourable response to immune checkpoint inhibition. However, in our study, seven of the eight patients were negative for PD-L1 expression. There are contradictory results for the prognostic value of PD-L1 expression in glioblastoma patients for survival outcomes. However, we observed that one PD-L1-positive patient also achieved a durable response to second-line therapy. It can be speculated that the PD-1/PD-L1 signalling pathway does not play a key role in the development of glioblastoma and may be influenced by other factors [20, 21]. Therefore, it is difficult to obtain satisfactory results by blocking the PD-1/PD-L1 pathway alone. Thus, we have implemented combination therapy. In the CheckMate 143 report, patients treated with nivolumab in combination with or without ipilimumab for recurrent glioma were divided into three groups according to the concentration of the drug used; the highest objective remission rate was 11%, and the longest median PFS rate was 2.1 months [22]. Another objective remission rate of 10.4% was reported for the combination of lysovirus and pembrolizumab [23]. Song LIn et al. reported an objective remission rate of 20% using a combination of radiation therapy and immunotherapy for recurrent glioma [24]. This is a good example of how immunotherapy can be used to treat recurrent glioma. Taken together, these findings demonstrate that the efficacy of immune therapy in combination with other therapies for recurrent glioma has improved. However, in our study, the ORR reached 62.5%, and one patient still had PR. Our longer ORR might be related to alteration of the immune microenvironment by chemotherapy and targeted drugs.

TMZ is valuable treatment for glioma. When considering which drug should be included in our combination regimen, TMZ was an obvious choice. Moreover, it has been shown that TMZ can promote immune escape in GBM cells by upregulating expression of PD-L1 [19]. Therefore, blocking the interaction between PD-1 and PD-L1 antibodies or inhibiting the PD-L1-induced pathway is an effective strategy for enhancing the immune response. The combination of PD-L1 and TMZ has been demonstrated in animal studies. Human studies have also shown that TMZ can be used to successfully deliver cellular immunotherapy. In addition, restoration of homeostatic lymphocytes after TMZ-induced lymphopaenia is a window for rapid expansion of antigen-specific T cells [25, 26]. TMZ can be used as an immunomodulator in GBM patients receiving immunotherapy.

Vascular endothelial growth factor (VEGF) is overexpressed in GBM. VEGF levels correlate directly with tumour vascularity and grade and negatively with prognosis [27]. Angiogenesis and immune tolerance are both normal physiologic mechanisms that are hijacked by tumours. Vascular endothelial growth factors drive immune suppression by directly inhibiting antigen-presenting cells and immune effector cells or by enhancing the function of regulatory T cells (Treg), myeloid-derived suppressor cells (MDSCs), and tumour-associated macrophages (TAMs). These inhibitory immune cells can, in turn, promote angiogenesis, creating a malign cycle that compromises immune activation. Hence, antiangiogenic and immunomodulatory effects appear to be bidirectional processes [28]. In additional, VEGF exerts an immunosuppressive activity by inducing down-regulation of antigen presentation through the inhibition of dendritic cell maturation. Hence, reducing the angiogenic pathways in the TME could increase antitumour immune response [29]. In the field of lung cancer, the combination of bevacizumab with immunosuppressive agents helps to overcome resistance to immune checkpoint inhibitors and increase the efficacy of immunotherapy [30]. The single-agent bevacizumab has been approved for treatment of recurrent GBM, but additional studies are focusing on the combination of antiangiogenic therapies with cytotoxic chemotherapeutic agents. A prospective and multicentre phase II study involving eight sites enrolled 32 patients previously treated with radiotherapy and at least three cycles of adjuvant TMZ and bevacizumab (BV) to assess the efficacy and safety of treatment for patients with recurrent glioblastoma (GB). The estimated 6-month PFS rate was 21.9% (95% CI 9.3–40.0%). The median PFS and overall survival (OS) were 4.2 months (95% CI 3.6–5.4 months) and 7.3 months (95% CI 5.8–8.8 months), respectively [31]. Another study of recurrent glioma enrolled 60 patients treated with bevacizumab in combination with TMZ; the median survival time was 4.7 months, and the ORR was 19.0% [32]. Our study showed a higher ORR but a lower mPFS, perhaps because four of our eight patients received the three-drug combination as a second-line regimen (two patients had been treated with bevacizumab monotherapy after their first relapse, and two patients entered the clinical trial to receive target-exempt therapy); additionally, cross-line use of bevacizumab may have contributed to this result. However, the immunomodulatory effects of TMZ and the bidirectional modulatory effects of antiangiogenesis agents and immunity might lead to a greater effect of immune drugs in combination, ultimately resulting in a higher objective remission rate.

In our study, the triple drug therapy was well tolerated, and although there was one case of a grade 3 adverse event, the patient’s symptoms improved after symptomatic medication and did not interfere with subsequent treatment. The study suffered from selection and sampling bias because it was a retrospective analysis of patients from a single institution. In addition, the small sample size makes it difficult to draw meaningful conclusions that can be generalized to larger populations. Furthermore, the sample of patients selected for this study was heterogeneous, with varying prior, concurrent, and subsequent treatments, which may have affected the efficacy of immunotherapy. As a small-cohort study, it was therefore not possible to prospectively assess T-cell microenvironmental alterations, and the limited number of responses made it challenging to assess the impact of biomarkers. Based on these findings, we believe that the true efficacy of TMZ + bevacizumab + sintilimab in treatment of recurrent glioma must be confirmed in a prospective trial, and we are currently conducting such a study (NCT05638451). Three patients have already been enrolled, and we look forward to reporting these data

## Data Availability

The retrospective data used to support the findings of this study are available from the corresponding author upon request.
